# ValLAI_Crop, a validation dataset for coarse-resolution satellite LAI products over Chinese cropland

**DOI:** 10.1038/s41597-021-01024-4

**Published:** 2021-09-20

**Authors:** Bowen Song, Liangyun Liu, Shanshan Du, Xiao Zhang, Xidong Chen, Helin Zhang

**Affiliations:** 1 Key Laboratory of Digital Earth Science, Aerospace Information Research Institute, Chinese Academy of Sciences, Beijing, 100094 China; 2 University of Chinese Academy of Sciences, Beijing, 100049 China; 3 State Key Laboratory of Remote Sensing Science, Jointly Sponsored by Beijing Normal University and Institute of Remote Sensing and Digital Earth of Chinese Academy of Sciences, Beijing, 100875 China; 4 Beijing Engineering Research Center for Global Land Remote Sensing Products, Institute of Remote Sensing and Engineering, Faculty of Geographical Science, Beijing Normal University, Beijing, 100875 China

**Keywords:** Environmental sciences, Carbon cycle

## Abstract

Numerous validation efforts have been conducted over the last decade to assess the accuracy of global leaf area index (LAI) products. However, such efforts continue to face obstacles due to the lack of sufficient high-quality field measurements. In this study, a fine-resolution LAI dataset consisting of 80 reference maps was generated during 2003–2017. The direct destructive method was used to measure the field LAI, and fine-resolution LAI images were derived from Landsat images using semiempirical inversion models. Eighty reference LAI maps, each with an area of 3 km × 3 km and a percentage of cropland larger than 75%, were selected as the fine-resolution validation dataset. The uncertainty associated with the spatial scale effect was also provided. Ultimately, the fine-resolution reference LAI dataset was used to validate the Moderate Resolution Imaging Spectroradiometer (MODIS) LAI product. The results indicate that the fine-resolution reference LAI dataset builds a bridge to link small sampling plots and coarse-resolution pixels, which is extremely important in validating coarse-resolution LAI products.

## Abstract (summary)

**Table:** 

Measurement(s)	leaf area index
Technology Type(s)	destructive sampling method
Sample Characteristic - Environment	area of cropland
Sample Characteristic - Location	China

Machine-accessible metadata file describing the reported data: 10.6084/m9.figshare.15124524

## Background & Summary

The leaf area index (LAI), defined as one-half of the total leaf area per unit ground surface area^1^, is a critical parameter used to characterize the structure and function of vegetation^2^. Since the LAI directly relates to the acquisition and utilization of sunlight by leaves, it is a key parameter in terrestrial ecosystem models and closely related to the carbon cycle as well as to photosynthesis, respiration and transpiration in leaves^3^.

Many global and regional LAI products with different temporal and spatial resolutions exist that are derived using various retrieval algorithms and can be applied in studies addressing ecophysiology, atmosphere-ecosystem interactions and global change^4,5^. However, due to the limitations resulting from radiometric calibration, the atmospheric correction of raw data, the scale effect, and retrieval algorithms, errors inevitably exist in satellite products. Thus, to make appropriate use of satellite products, it is essential to investigate and quantify the uncertainties associated with these products^6,7^.

Field measurements serve as ‘reference’ values and constitute an important part of the validation of remote sensing products^8,9^. LAI measurement methods are generally categorized into direct and indirect methods^10^. Indirect methods include optical methods based on Beer’s law and inclined-point quadrat methods, in which the LAI is calculated by measuring other variables, such as the gap fraction, light transmission, and the contact number. However, the influences of the clumping effect, woody components and the leaf angle distribution (LAD) also need to be considered^11–13^. However, correcting for these variables is challenging because difficulties in their accurate measurment^14^. Several methods have been developed to correct the clumping index, including the finite-length averaging method^15^, the gap-size distribution method^16,17^, a combination of the gap-size distribution and finite-length averaging methods^18^, and the path length distribution method^11^. These methods, which have been applied for decades, should increase accuracy and be able to be used for new applications. Many comparisons of direct and indirect methods of LAI measurement for crops and forests have also been made^19,20^. The results of these comparisons have indicated that the indirect methods can underestimate the LAI, which may be due to the clumping of branches and stems, especially in forested areas^19,21^. In the case of corn, the indirect measurements made by the AccuPAR ceptometer, which measures photosynthetically active radiation (PAR) and inverts these readings to acquire canopy LAI, were shown to give higher mean values of LAI than those collected using destructive methods^22,23^. Moreover, with the exception of techniques such as downwards-facing digital hemispherical photography (DHP)^24,25^, it is very challenging to measure the LAI of low vegetation, such as wheat and paddy rice in the early growth stages, using indirect optical methods due to difficulties in collecting the downward radiation through canopies.

In direct methods, plants are collected in a destructive way, and the LAI is determined by measuring the area of the sampled leaves and then dividing by the sampling area. Values of the LAI determined using direct methods are thus considered to be the most accurate^4^, and they are therefore used to calibrate indirect measurements. Although direct methods are much more time consuming and labour intensive than indirect optical-instrument methods, the use of destructive direct measurement methods is still feasible for small samples of low vegetation such as crops.

In general, an individual pixel of a satellite product covers a certain range on the ground that does not match the area represented by the sampling point on the ground. To overcome the spatial mismatch between field measurements and coarse-resolution LAI products^26^, multiscale validation based on fine-resolution satellite or airborne remote sensing imagery is employed to bridge the gap between ground measurements and coarse-resolution satellite data. Several previous validation efforts have partially addressed the scale problem in remote sensing: for example, the Bigfoot^27^ program links field measurements and Landsat-7 ETM data to generate high spatial-resolution maps to overcome spatial mismatch, and in the Cold Land Processes Field Experiment^28^ (CLPX), a multiscale dataset based on a nested sampling strategy for upscaling was built. A series of protocols and good practices for the validation of global LAI products have been established by the CEOS/WGCV LPV subgroup^29^. The strategy of validation proposed by the CEOS/WGCV LPV subgroup consists of direct validation and intercomparison approaches^2,30,31^. The On Line Interactive Validation Exercise (OLIVE)^32^ platform hosted by the European Space Agency (ESA) Cal/Val portal followed the guidelines of CEOS/WGCV LPV and provided two independent datasets for validation: BELMANIP2^33^ and DIRECT^2^, which contained 445 and 113 sites, respectively. The Validation of Land European Remote sensing Instruments (VALERI) project focuses on validation activity to obtain consistent approaches and acquire data in a synergistic way. For the purpose of validating coarse-resolution satellite products, this validation project has developed high spatial-resolution (10–30 m) maps of biophysical variables including LAI that were calibrated using ground measurements^34^. In the FP7 ImagineS project, field measurements have been collected to evaluate the products of the Copernicus Global Land Service (CGLS) derived from satellites since 2013, and the *in situ* measurements were processed according to the guidelines defined by the CEO/WGCV LPV subgroup^31^. High spatial-resolution imagery was employed to upscale the local measurements by EOLAB to generate reference maps of LAI based on the protocols established by the VALERI project^35^. In addition, many validation efforts have also been carried out in China. The Heihe Integrated Observatory Network was established for long-term observations in 2007. Additionally, a series of multiscale observation experiments over heterogeneous land surfaces were conducted in the Heihe River Basin (HRB)^36^. Among those long-term observations in the HRB, the Heihe Watershed Allied Telemetry Experimental Research (HiWATER) team investigated the LAI on the basis of regular manual observation during 2013–2015^37^, and automatic observation devices for monitoring the LAI were installed and have been operational at three superstations since 2018. In addition, a seasonal field campaign was carried out by Fang *et al*. to collect LAI measurements of paddy rice, maize, soybean and sorghum using indirect optical methods in Northeast China in 2012–2013 and 2016, which were used to evaluate satellite-based LAI products^38,39^.

Direct validation of coarse-resolution LAI products derived from remote sensing data works in concert with the comparison of satellite products with upscaled field LAI maps on the basis of spatial–temporal synchronization^40^. Numerous efforts have been conducted to validate coarse-resolution LAI products using fine-resolution LAI maps calibrated with *in situ* measurements^24,25,41–43^. Unfortunately, almost all the validation datasets for coarse-resolution LAI products are based on indirect field measurements, and the uncertainties in these data could be transferred to the products to be validated. Various conclusions regarding the validation of LAI products over croplands have thus far been drawn. Early studies found that the Moderate Resolution Imaging Spectroradiometer (MODIS) LAI generally underestimates the LAI of crops at the senescence stage^35,44^. By evaluating the GLASS, MODIS (V6), and VIIRS products, Fang *et al*. (2019) found that the LAI was underestimated at a paddy rice site, especially when LAI > 3.0; the results also indicated an overestimation of GEOV2 for rice^39^. However, Campos-Taberner *et al*. (2018) recently presented results showing that GEOV1, MODIS (V5) and EPS performed well for rice in southern Europe (root-mean-square error (RMSE) ≤ 0.80)^45^. Luke *et al*. (2020) assessed the CGLS 300 m V1, MODIS (V6), and VIIRS (V6) LAI products in North America, and the results indicated that the CGLS 300 m V1 gave the best agreement (root-mean-square deviation (RMSD) = 0.57) in comparison with RMSD values of 0.81 and 0.89 for VIIRS and MODIS (V6) products, respectively^43^. Xu *et al*. (2018) assessed the uncertainties/relative uncertainties of VIIRS and MODIS LAI products using ground measurements, with observed values of 0.60/42.2% and 0.55/39.3%, respectively^46^.

The accurate and comprehensive validation of coarse-resolution LAI products is still very difficult due to the lack of sufficient direct field measurements. The aim of this study was to develop a highly accurate LAI validation dataset with fine-resolution for Chinese croplands to validate coarse-resolution satellite products based on direct field measurements. The fine-resolution reference LAI maps were generated from Landsat imagery using a local semiempirical model; these maps were then used as a bridge to evaluate the coarse-resolution products. Here, reference maps were applied to validate the accuracy of the MODIS LAI product.

## Methods

### Study area

**Fig. 1:**
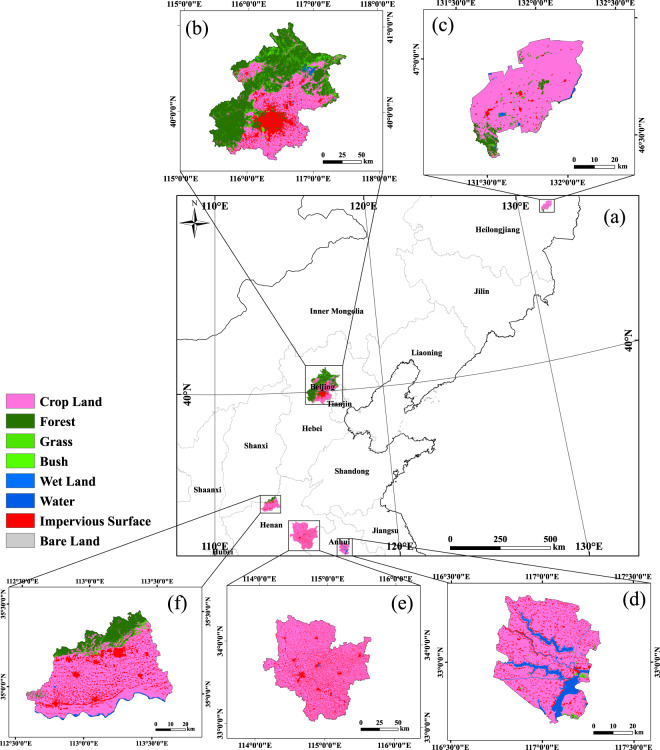
(**a**) Location and vegetation type of the study areas in (**b**) Beijing, (**c**) Youyi Farm in Heilongjiang Province, (**d**) Longkang Farm in Anhui Province, (**e**) Zhoukou, Henan Province, and (**f**) Jiaozuo, Henan Province.

Field LAI measurements were collected in four areas: Beijing, Henan Province, Heilongjiang Province, and Anhui Province, as illustrated in Fig. 1. Online-only Table 1 shows detailed information about the field measurements and selected Landsat surface reflectance images in the four study areas. A total of 1010 samples corresponding to 43 growth stages were collected during the experiments. The collected samples included wheat, barley, paddy rice and soybean. The specific sampling dates, numbers of samples, and types of crops are listed in Online-only Table 1.

The experiments in Beijing were carried out during the winter wheat growing seasons from 2004 to 2007. Beijing is located in the north of the North China Plain, which is a warm temperate zone with a semihumid and semiarid monsoon climate.

The study sites in Henan Province were located in Jiaozuo and Zhoukou, which have temperate monsoon climates with abundant sunshine and a clear difference between the summer and winter temperatures. The average annual temperature in these areas is between 12.8 °C and 14.8 °C. The annual average precipitation is 644.3 mm, with 45%–60% of the precipitation falling from June to August. The crop grown at these study sites is winter wheat.

The study area in Heilongjiang Province was located at Youyi Farm, which is situated on the Sanjiang Plain. The total cultivated area of this study area is 1104.29 km^2^, and the main crops are wheat, barley, paddy rice and soybean. The region has a temperate continental monsoon climate with a mean annual temperature of 3.4 °C. The annual average precipitation is approximately 540 mm, and the precipitation is concentrated in the summer. The Sanjiang Plain is one of the most well-known black soil plains worldwide and is characterized by a low soil albedo.

The fourth field experiment was conducted at Longkang Farm (33°06′45.2″N, 116°51′44.8″E), Anhui Province, in 2017. This study area is located in the southern part of the Huaibei Plain. The study area has an elevation of approximately 22.7–25.9 m above sea level and covers a cultivated area of approximately 20 km^2^. It is located in a transition zone between the subtropics to the south and the warm temperate zone to the north. The site itself lies in the warm temperate semihumid monsoon agricultural zone and receives moderate rainfall and sufficient sunshine. The annual average amount of sunshine is approximately 2000 hours, which is approximately 54% of the possible maximum. The annual average temperature is 14.84 °C, and the average annual precipitation is approximately 789 mm.

### LAI measurements

All of the field LAI measurements were collected using a destructive sampling method. The locations of sampling points and vegetation types of study areas were illustrated in Figure S1 in the Supplementary Information. Plant samples were taken from areas of 1 m × 1 m; after being cut, they were quickly taken to the laboratory. All of the fresh leaves were quickly weighed, and 10 typical leaves were scanned to determine the leaf area. These 10 typical leaves and the remaining leaves were then dried in an oven until a constant weight (the dry weight, DW) was reached so that the leaf DW could be obtained. The specific leaf weight (SLW) and LAI were determined as follows:

1 $${\rm{SLW}}=\frac{{\left({\rm{DW}}\right)}_{0}}{{{\rm{A}}}_{0}}\left(g.c{m}^{-2}\right)$$

2 $${\rm{LAI}}=\frac{{\rm{DW}}}{{\rm{SLW}}\times {{\rm{A}}}_{{\rm{s}}}}$$

where DW is the total dry weight of the leaves; A_0_ and (DW)_0_ are the area and dry weight of the typical leaves, respectively, which were used to calculate the SLW; and A_s_ is the sampling area (1 m × 1 m). Here, the elementary sampling unit (ESU) method^29,31^ was not employed to collect LAI measurements due to the large amount of effort required to implement the destructive method. The crops were relatively uniform in comparison to the natural vegetation. According to investigations by Song *et al*.^47^, the spatial heterogeneity of winter wheat is relatively small, with a variation coefficient less than 6% for the optimized soil-adjusted vegetation index (OSAVI). Thus, only one uniform plot with a size of 1 m × 1 m was sampled to represent a Landsat TM pixel. In addition, more than 20 samples were collected to build a semiempirical model to retrieve the LAI in each growth stage, with which a fine-resolution LAI map can be generated.

### Landsat surface reflectance data and normalization

The Landsat-5 TM and Landsat-8 OLI surface reflectance (SR) products, for which a sufficient number of satellite images acquired at the same time as the field measurements were available, were used as a ‘bridge’ for upscaling the field LAI measurements to match the coarse-resolution LAI products. All of the Landsat TM and OLI SR images were downloaded from the United States Geological Survey (USGS) EarthExplorer website (https://earthexplorer.usgs.gov). All of these data consisted of SR products that had been derived from Level-1 data by atmospheric correction. Landsat TM/ETM SR data are generated with specialized software called the Landsat Ecosystem Disturbance Adaptive Processing System (LEDAPS)^48^. Landsat-8 OLI SR data are generated from the Land Surface Reflectance Code (LaSRC), which makes use of the coastal aerosol band to perform aerosol inversion tests and uses MODIS auxiliary climate data and a unique radiative transfer model^49^. The criteria for the selection of the Landsat SR images were that the imagery should be cloud free and acquired within seven days of the field measurements^50^. As a result, a total of eight Landsat-5 TM imagers that matched the field measurements (path 123/row 32) were collected for the Beijing area. For Henan Province, four clear Landsat-5 TM images of the Zhoukou area (path 123/row 37) and one Landsat-5 TM image of the Jiaozuo area (path 125/row 36) that matched the field measurements were found. Five clear Landsat-5 TM images (path 114/row 28, path 115/row 27) that covered Youyi Farm, Heilongjiang Province, and two clear Landsat-8 images of Longkang Farm, Anhui Province, were selected. The acquisition dates of the satellite imagery are listed in Online-only Table 1. Because of the limitations on the observation time and degree of cloud contamination in the Landsat satellite imagery, data from only 20 of the 43 field experiments listed in Online-only Table 1 were used to generate the fine-resolution reference LAI maps.

The satellite-based NDVI is a crucial variable in the semiempirical model during the upscaling procedure. To reduce the uncertainty related to the data quantification and determine the parameters in semiempirical models more accurately, the Landsat-5 TM SR imagery was normalized using the MODIS (MCD43A4) version 6 Nadir Bidirectional Reflectance Distribution Function (BRDF)-Adjusted Reflectance (NBAR) product^51^, which provides 500 m reflectance data adjusted using a bidirectional reflectance distribution function to model the reflectance values as if they were taken at nadir view.

Relative radiation normalization is widely used to eliminate the radiation differences among images acquired at different epochs or collected by different space-borne instruments. A clear SR image was generally selected as a reference to normalize the target image using a linear regression model band by band^52^. Here, it was employed to normalize the Landsat TM SR image using the MODIS SR data as a reference. To obtain the linear regression model for normalization processing, the 30 m TM images were aggregated to a resolution of 500 m and converted to the same sinusoidal projection as the MODIS product used; then, linear regression models were built to link Landsat TM data to MODIS SR data band by band. If the determination coefficient (R^2^) was greater than 0.75, the TM SR data were normalized using the linear regression model; otherwise, the ratio of the mean values of the TM and MODIS SR data was used to normalize the Landsat TM SR data.

**Fig. 2:**
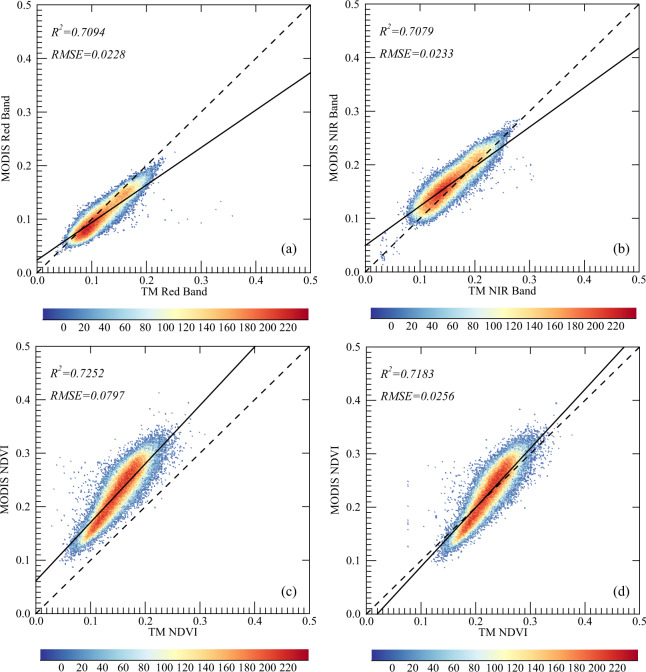
Normalization of the Landsat-5 TM SR data using the MCD43A4 product for the Beijing study area on April 1, 2004. (**a**) Plot of TM red-band reflectance against MODIS red-band reflectance; (**b**) plot of TM near-infrared reflectance against MODIS near-infrared reflectance; (**c**) correlation between MODIS and TM NDVI values before calibration; (**d**) correlation between MODIS and TM NDVI values after calibration.

A comparison of the MODIS and Landsat TM SR products (including the reflectance at the red and near-infrared bands and the NDVI) was therefore performed to normalize the Landsat SR products. Figure 2 shows the scatterplot of the normalized Landsat SR product data against the MCD43A4 data on April 1, 2004, in Beijing. The results show that the regression lines deviate from the 1:1 line, indicating that the TM red-band reflectance was higher than that of the MODIS data and that the Landsat NDVI values were smaller than the corresponding MODIS values. The normalization functions for Landsat TM red and near-infrared bands in the Beijing, Henan, and Heilongjiang study areas are illustrated in Tables S1–S3 in the Supplementary Information. The corresponding scatterplots are also provided in Figures S2–S7 in the Supplementary Information.

### MODIS LAI product (MCD15A2H)

In this study, we applied the fine-resolution validation dataset to assess the MODIS LAI product with coarse-resolution, one of the most commonly used global LAI products. The MODIS LAI product version 6 (MCD15A2H) was devised by Myneni *et al*.^53^ in 2015. This product is widely known as a mainstream global LAI product and has been applied to the modelling of atmospheric carbon assimilation, crop growth, and evapotranspiration. It is produced using a combination of Terra and Aqua data acquired every 8 days at a 500-m spatial resolution. The algorithm used to produce this product is based on three-dimensional radiative transfer theory, which is ultimately optimized using a look-up table (LUT) to solve the radiative transfer equation^54^. In addition to the main LUT method, a back-up algorithm based on directional vegetation indices can be employed to retrieve the LAI for different biomes^55^.

### Semiempirical NDVI-based model for generating fine-resolution LAI validation maps

**Fig. 3:**
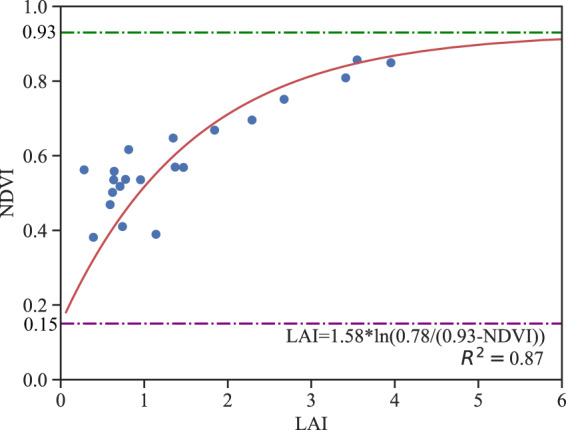
Relationship between NDVI and LAI using measurements of winter wheat in Beijing. NDVI_∞_ represents the asymptotic value of NDVI when LAI tends towards infinity, NDVI_bs_ represents the NDVI value corresponding to that of the bare soil, and K_ndvi_ is the extinction coefficient in the NDVI-based LAI model.

A semiempirical model was employed to model the relationship between the NDVI and LAI. This model was based on the Beer-Lambert Law^56^:

3 $$NDVI=NDV{I}_{\infty }+(NDV{I}_{bs}-NDV{I}_{\infty })\ast exp(-{K}_{ndvi}\ast LAI)$$

where *NDVI*_*bs*_ is the NDVI value of bare soil, *NDVI*_*∞*_ is the NDVI value corresponding to saturation of the LAI, and *K*_*ndvi*_ is the extinction coefficient, which is related to the structure of the scattering community (in particular, the leaf inclination distribution) and the leaf optical properties. The parameters in Eq. (3) were optimized to produce the best accuracy for the Landsat scenes covering the different study areas using the local experimental data at different growth stages and a curve-fitting algorithm to give the lowest fitting error^57^. For instance, *NDVI*_*∞*_ = 0.93, *NDVI*_*bs*_ = 0.15 and *K*_*ndvi*_ = 1.58 were derived from the experimental data obtained on April 1, 2004, in Beijing, as illustrated in Fig. 3.

Once the parameters in Eq. (3) had been determined using the field data, the NDVI-based regression model could be used to generate the fine-resolution LAI maps using the equation

4 $$LAI={K}_{ndvi}\ast ln\left((NDV{I}_{\infty }-NDV{I}_{bs})/\left(NDV{I}_{\infty }-NDVI\right)\right)$$

The fine-resolution 30 m LAI maps were first generated using Landsat SR images for different growth stages and areas using the appropriate NDVI-based model. Cloud-free reference LAI maps with a size of 3 km × 3 km centred on the field sampling points were then acquired for use as potential validation maps. Finally, the proportion of cropland in each 3 km × 3 km reference map was calculated using the GLOBELAND30–2010 land cover product^58^, as shown in Fig. 1. Only the potential LAI validation maps with a proportion of cropland larger than 75% were selected for use as validation maps.

### LOOCV validation method

Due to limited field measurements in each growth stage, the leave-one-out cross-validation (LOOCV) approach^59^ and curve-fitting algorithm were employed to generate the NDVI-based LAI model. The LOOCV method splits a dataset into a training set and a testing set using all but one observation as part of the training set. For example, there were 22 samples in the Beijing field experiment performed on April 1, 2004. The LOOCV approach chose 21 observations as training samples and one observation as a validation sample. This procedure was repeated 22 times. For each repeat, 21 field measurements were used to determine the parameters in Eq. (4) based on the curve-fitting algorithm. This algorithm is in the Python scipy.optimize module, which uses nonlinear least squares to fit a function^57^. Due to the limitation of sample size, we were required to set the bounds for the parameters, and the algorithm derives the optimal values for the parameters through iteration so that the sum of the squared residuals of the function is minimized. The value range of NDVI_∞_ is 0.91–0.97, NDVI_bs_ ranged between 0.01 and 0.18, and K_ndvi_ is in the range of 1.3–1.8. Thus, 22 statistical equations were obtained during the procedure. All the field measurements were separately brought into the 22 equations to identify the equation with the lowest RMSE, which was selected as the equation to generate the fine-resolution LAI map.

The equations used to generate the fine-resolution LAI map for each growth stage in the different study areas are shown in Table 1.

**Table 1:** NDVI-based statistical models used to generate fine-resolution LAI at each growth stage in the different study areas.

Date	Model	Site
2004/4/1	LAI = 1.58*ln(0.78/(0.93-NDVI))	BJ-wheat
2004/4/17	LAI = 1.49* ln (0.96/(0.97-NDVI))	
2004/5/19	LAI = 1.44* ln (0.96/(0.97-NDVI))	
2005/4/4	LAI = 1.30*ln(0.86/(0.97-NDVI))	
2005/5/6	LAI = 1.46*ln(0.834/(0.93-NDVI))	
2005/5/22	LAI = 1.42*ln(0.96/(0.97-NDVI))	
2006/4/7	LAI = 1.36* ln (0.96/(0.97-NDVI))	
2007/4/10	LAI = 1.48* ln (0.96/(0.97-NDVI))	
2003/3/30	LAI = 1.58*ln(0.96/(0.97-NDVI))	HN-wheat
2004/4/8	LAI = 1.66*ln(0.96/(0.97-NDVI))	
2004/4/24	LAI = 1.62*ln(0.96/(0.97-NDVI))	
2004/5/10	LAI = 1.59*ln(0.91/(0.96-NDVI))	
2004/5/17	LAI = 1.44*ln(0.76/(0.91-NDVI))	
2005/05/23	LAI = 1.30*ln(0.81/(0.95-NDVI))	HLJ-barley
2006/06/02	LAI = 1.30*ln(0.944/(0.97-NDVI))	
2007/06/14	LAI = 1.36*ln(0.90/(0.92-NDVI))	
2005/05/23	LAI = 1.30*ln(0.822/(0.94-NDVI))	HLJ-wheat
2006/06/02	LAI = 1.30*ln(0.858/(0.96-NDVI))	
2017/3/29	LAI = 1.33*ln(0.9400/(0.95-NDVI))	AH-wheat
2017/4/23	LAI = 1.80*ln(0.9505/(0.96-NDVI))	

Several quality indicators were employed to assess the reference maps and LAI products, including the RMSE, relative root mean square error (RRMSE), coefficient of determination (R^2^), and relative bias. Relative bias is the relative difference between the corresponding reference LAI and field LAI. It was defined as follows:

5 $${\rm{Relative}}\;{\rm{bias}}=(mea{n}_{LA{I}_{ref}}-mea{n}_{LA{I}_{field}})/mea{n}_{LA{I}_{field}}$$

where $mea{n}_{LA{I}_{ref}}$ represents the mean value of the estimated reference LAI in each growth stage and $mea{n}_{LA{I}_{field}}$ represents the mean value of the field LAI in each growth stage.

Uncertainty is one of most important indicators used to represent the accuracy of reference maps and is of great significance for product validation. The uncertainty was defined as follows:

6 $${\rm{Uncertainty}}={{\rm{LAI}}}_{{\rm{mean}}}\times {\rm{RRMSE}}$$

where LAI_mean_ represents the mean value of LAI within the 3 km × 3 km reference map and RRMSE represents the relative root mean square error between the generated and field-measured LAI in each growth stage.

### Determination of scaling difference using different upscaling methods

In the absence of scaling errors, Tian *et al*. (2003) found that the LAI obtained from coarse-resolution satellite data should be equal to the arithmetic average of values obtained from fine-resolution data^60^. Due to the heterogeneity of the land surface and nonlinearity of the inversion model, scaling errors are inevitable in retrieving LAI at coarse spatial resolution^61–63^. To investigate the scaling errors inherent to the coarse-resolution LAI product, the differences in the U1 and U2 upscaling methods were obtained to partly quantify the errors in product validation. The upscaling method U1 is the so-called ‘invert first and then average’ method, in which the fine-resolution NDVI is calculated first and the fine-resolution LAI is then retrieved based on the semiempirical NDVI-based model. The fine-resolution LAI maps are then aggregated (i.e., upscaled) to generate the coarse-resolution LAI. The upscaling method U2 is the so-called ‘average first and then invert’ method. Using this method, the fine-resolution SR image is aggregated to a coarse-resolution image to derive the coarse-resolution NDVI. The semiempirical NDVI-based model is then used to retrieve the coarse-resolution LAI. The difference in pixel value between the coarse-resolution LAI images obtained using the two different upscaling methods can be regarded as the spatial-scale difference^26,61^. Details regarding scaling differences are provided in the Supplementary Information.

## Data Records

On the basis of the selection rules introduced in the **Semiempirical NDVI-based model for generating fine-resolution LAI validation maps** section, a total of 80 fine-resolution LAI validation maps with a size of 3 km × 3 km were generated from the Landsat-5 TM and Landsat-8 OLI reflectance data; these maps are provided in the Supplementary Information, Figures S9–S13. Detailed statistical metrics for these 80 fine-resolution maps are summarized in Tables 2–5.

**Table 2:** Statistical metrics of the fine-resolution LAI maps of the Beijing study area.

ID	Date	Latitude (°)	Longitude (°)	Crop cover	Mean_LAI_U1	Uncertainty	Standard deviation	Mean_LAI_U2	Scaling difference
1	2004/4/1	40.22732	116.81093	0.94	0.439	0.117	0.229	0.422	0.017
3	2004/4/1	40.20461	116.35767	0.86	0.450	0.121	0.335	0.428	0.022
12	2004/4/1	40.17907	116.56123	0.93	0.505	0.135	0.313	0.548	−0.042
13	2004/4/1	39.766	116.71623	0.90	0.484	0.130	0.266	0.457	0.028
1	2004/4/17	40.2273	116.81019	0.94	0.916	0.230	0.790	0.785	0.130
4	2004/4/17	40.20432	116.358	0.87	1.009	0.253	0.869	1.003	0.006
10	2004/4/17	40.17932	116.5612	0.93	1.111	0.279	0.939	1.191	−0.080
13	2004/4/17	39.76626	116.71829	0.90	0.793	0.199	0.493	0.799	−0.006
1	2004/5/19	40.22867	116.81305	0.94	1.108	0.230	0.772	1.044	0.064
3	2004/5/19	40.20434	116.35803	0.87	1.275	0.265	1.049	1.659	−0.384
11	2004/5/19	40.17934	116.56123	0.93	1.292	0.269	0.935	1.315	−0.023
14	2004/5/19	39.76601	116.71798	0.90	0.943	0.196	0.623	0.895	0.048
8	2005/4/4	40.17293	116.58101	0.96	0.467	0.207	0.294	0.404	0.063
14	2005/4/4	39.76601	116.71658	0.90	0.293	0.130	0.171	0.313	−0.020
15	2005/4/4	39.73602	116.72371	0.91	0.325	0.144	0.126	0.299	0.026
17	2005/4/4	39.67929	116.73827	0.89	0.273	0.121	0.145	0.271	0.002
8	2005/5/6	40.17293	116.58101	0.96	1.899	0.403	1.347	1.730	0.169
14	2005/5/6	39.76601	116.71658	0.90	1.311	0.278	1.052	1.264	0.047
15	2005/5/6	39.73602	116.72371	0.91	2.223	0.471	1.166	1.936	0.286
17	2005/5/6	39.67929	116.73827	0.89	1.782	0.378	1.408	1.525	0.257
8	2005/5/22	40.17293	116.58101	0.96	2.072	0.531	1.137	1.905	0.167
14	2005/5/22	39.76601	116.71658	0.90	1.435	0.367	0.909	1.463	−0.028
15	2005/5/22	39.73602	116.72371	0.91	2.257	0.578	0.987	2.033	0.223
17	2005/5/22	39.67929	116.73827	0.89	1.711	0.438	1.034	1.543	0.169
8	2006/4/7	40.17102	116.57608	1.00	1.125	0.350	0.794	1.045	0.079
15	2006/4/7	39.76628	116.71763	0.90	0.816	0.254	0.404	0.792	0.024
16	2006/4/7	39.73629	116.7237	0.91	1.160	0.361	0.508	1.105	0.055
18	2006/4/7	39.67902	116.73828	0.89	0.962	0.299	0.544	0.902	0.060
2	2007/4/10	40.17182	116.5722	1.00	1.433	0.385	0.914	1.408	0.025
14	2007/4/10	39.76628	116.71938	0.90	1.104	0.297	0.569	1.137	−0.033
18	2007/4/10	39.73575	116.72406	0.91	1.792	0.482	0.985	1.838	−0.046
20	2007/4/10	39.67794	116.73828	0.89	1.461	0.393	1.015	1.303	0.157

The mean LAI is the average LAI within each 3 km × 3 km reference map. The uncertainty is the product of the mean LAI and the RRMSE obtained using the NDVI-based inversion model. The standard deviation represents the spatial heterogeneity of the fine-resolution LAI maps. The scaling difference is the difference between the mean LAI values generated using the two different upscaling methods. The IDs correspond to the file names for the reference LAI maps.

**Table 3:** Statistical metrics of the fine-resolution LAI maps of the study areas in Jiaozuo and Zhoukou, Henan Province.

ID	Date	Latitude (°)	Longitude (°)	Crop cover	Mean_LAI_U1	Uncertainty	Standard deviation	Mean_LAI_U2	Scaling difference
5	2003/3/30	33.81258	114.63295	0.86	2.451	0.569	0.952	2.349	0.102
11	2003/3/30	33.74116	114.42932	0.88	2.228	0.517	0.980	2.406	−0.178
14	2003/3/30	33.73983	114.69064	0.96	3.202	0.743	1.214	2.855	0.347
16	2003/3/30	33.71973	114.37526	0.93	3.207	0.744	1.181	3.454	−0.247
2	2004/4/8	35.14029	113.02383	0.93	2.561	0.336	1.770	1.758	0.803
6	2004/4/8	35.11618	112.99328	0.96	2.733	0.358	1.721	2.741	−0.008
8	2004/4/8	34.95611	112.76271	0.83	3.604	0.472	2.053	2.820	0.784
11	2004/4/8	34.93798	112.99126	0.79	3.567	0.467	1.981	2.774	0.793
2	2004/4/24	35.14029	113.02383	0.93	3.168	0.215	1.777	2.455	0.712
6	2004/4/24	35.11807	112.99333	0.95	3.436	0.234	1.571	3.608	−0.172
8	2004/4/24	34.95611	112.76271	0.83	3.746	0.255	1.547	3.267	0.479
11	2004/4/24	34.93798	112.99126	0.79	2.641	0.180	0.951	2.339	0.302
2	2004/5/10	35.14029	113.02383	0.93	3.118	0.243	1.401	2.560	0.558
6	2004/5/10	35.11706	112.98935	0.95	4.310	0.336	1.843	4.154	0.155
7	2004/5/10	34.95611	112.76271	0.83	3.607	0.281	1.331	3.287	0.320
10	2004/5/10	34.93798	112.99126	0.79	2.892	0.226	0.960	2.587	0.305
2	2004/5/17	35.14029	113.02383	0.93	2.347	0.305	1.295	1.776	0.570
6	2004/5/17	35.11837	112.99169	0.95	2.448	0.318	1.123	2.411	0.037
8	2004/5/17	34.95611	112.76271	0.83	2.059	0.268	0.902	1.878	0.181
11	2004/5/17	34.93659	112.99319	0.76	1.615	0.210	0.669	1.426	0.189

The mean LAI is the average LAI within each 3 km × 3 km validation reference map. The uncertainty is the product of the mean LAI and the RRMSE obtained using the NDVI-based inversion model. The scaling difference is the difference between the mean LAI values generated using the two different upscaling methods. The standard deviation represents the spatial heterogeneity of the fine-resolution LAI maps. The IDs correspond to the file names for the reference LAI maps.

**Table 4:** Statistical metrics of the fine-resolution LAI maps of the Youyi Farm study area, Heilongjiang Province.

ID	Date	Latitude (°)	Longitude (°)	Crop cover	Mean_LAI_U1	Uncertainty	Standard deviation	Mean_LAI_U2	Scaling difference
3	2005/5/23	46.78729	131.88652	1.00	0.763	0.269	0.437	0.835	−0.073
4	2005/5/23	46.78593	131.97661	0.85	0.466	0.165	0.236	0.517	−0.051
14	2005/5/23	46.75035	131.83915	0.92	0.375	0.132	0.190	0.328	0.047
19	2005/5/23	46.70117	131.86714	0.98	0.293	0.103	0.120	0.282	0.011
3	2006/6/2	46.77431	131.74556	0.88	0.702	0.100	0.393	0.689	0.012
4	2006/6/2	46.76551	131.8512	0.98	0.812	0.116	0.456	0.806	0.006
8	2006/6/2	46.74023	131.75914	0.94	0.812	0.116	0.488	0.735	0.077
13	2006/6/2	46.71375	131.715	1.00	0.654	0.093	0.413	0.555	0.098
1	2007/6/14	46.80207	131.80545	0.96	1.135	0.280	0.650	1.206	−0.071
2	2007/6/14	46.79537	131.89549	1.00	1.338	0.331	0.772	1.516	−0.178
3	2007/6/14	46.79412	131.75324	0.84	1.202	0.297	0.762	1.303	−0.101
16	2007/6/14	46.72528	131.89951	0.97	1.312	0.324	0.845	0.957	0.355
8	2005/5/23	46.79189	131.90669	0.91	0.709	0.274	0.355	0.651	0.058
14	2005/5/23	46.7641	131.74208	0.96	0.505	0.195	0.306	0.481	0.024
15	2005/5/23	46.75895	131.84729	0.97	0.438	0.169	0.213	0.481	−0.043
16	2005/5/23	46.73658	131.71468	0.93	0.492	0.190	0.319	0.400	0.092
1	2006/6/2	46.96788	131.97277	1.00	0.521	0.153	0.327	0.531	−0.009
2	2006/6/2	46.96206	131.98901	0.97	0.569	0.167	0.322	0.600	−0.031
3	2006/6/2	46.93895	131.97432	1.00	0.565	0.166	0.328	0.524	0.041
6	2006/6/2	46.89826	131.98191	1.00	0.483	0.141	0.323	0.494	−0.011

The mean LAI is the average LAI within each 3 km × 3 km reference map. The uncertainty is the product of the mean LAI and the RRMSE obtained using the NDVI-based inversion model. The scaling difference is the difference between the mean LAI values generated using the two different upscaling methods. The standard deviation represents the spatial heterogeneity of the fine-resolution LAI maps. The IDs correspond to the file names for the reference LAI maps.

**Table 5:** Statistical metrics of the fine-resolution LAI maps of the Longkang Farm study area, Anhui Province.

ID	Date	Latitude (°)	Longitude (°)	Crop cover	Mean_LAI_U1	Uncertainty	Standard deviation	Mean_LAI_U2	Scaling difference
1	2017/3/29	33.151	116.772	0.95	4.651	1.079	2.040	2.833	1.818
3	2017/3/29	33.116	116.804	0.98	4.435	1.029	1.511	3.980	0.455
5	2017/3/29	33.100	116.865	0.82	2.190	0.508	1.423	1.925	0.265
12	2017/3/29	33.087	116.899	0.99	2.312	0.536	1.324	1.987	0.325
1	2017/4/23	33.151	116.772	0.95	4.055	0.657	1.730	3.474	0.581
3	2017/4/23	33.116	116.804	0.98	4.457	0.722	1.235	4.141	0.316
8	2017/4/23	33.100	116.865	0.82	2.665	0.432	1.613	2.285	0.379
15	2017/4/23	33.087	116.899	0.99	3.200	0.518	1.352	2.919	0.281

The mean LAI is the average LAI within each 3 km × 3 km reference map. The uncertainty is the product of the mean LAI and the RRMSE obtained using the NDVI-based inversion model. The scaling difference is the difference between the mean LAI values generated using the two different upscaling methods. The standard deviation represents the spatial heterogeneity of the fine-resolution LAI maps. The IDs correspond to the file names for the reference LAI maps.

The scaling difference was taken as the difference between the mean LAI values generated using the two different upscaling methods that were introduced in Figure S8 in the Supplementary Information. The standard deviation reflects the spatial heterogeneity of the 3 km × 3 km fine-resolution LAI maps. The underestimation caused by the scaling difference for the Henan, Beijing, and Anhui study areas (which have relatively light soil substrates) and the overestimation for the Heilongjiang study area (where the soil background is dark) agree with the results of the investigation performed by Liu *et al*. (2014) and Chen *et al*. (2002), who found that there was an “underestimation for mixed pixels with bright non-vegetation components and an overestimation for those with dark non-vegetation components ”^26,64^.

Table 2 lists the statistical metrics of the fine-resolution LAI validation maps for Beijing. A total of 32 reference maps corresponding to eight growth stages were used between 2004 and 2007. The LAI for the 32 reference maps is relatively low, ranging from 0.273 to 2.257, with a mean uncertainty of 0.290. The spatial heterogeneity is relatively large and has a mean standard deviation of 0.720, which gives a relatively large scaling difference with a mean value of 0.046.

Table 3 lists the statistical metrics of the fine-resolution LAI validation maps in the study areas of Henan Province. Twenty reference maps corresponding to five growth stages were used from 2003 to 2004. The LAI for these 20 reference maps varies from 1.615 to 4.310, with a mean uncertainty of 0.364. The spatial heterogeneity is higher than that for the Beijing study area and has a mean standard deviation of 1.361. The scaling difference is still obvious and has a mean value of 0.302.

Table 4 lists the statistical metrics of the fine-resolution LAI validation maps for Youyi Farm, Heilongjiang Province. Here, 20 reference maps corresponding to five growth stages were used from 2005 to 2006. The LAI in these maps is relatively low, ranging from 0.293 to 1.338, with a mean uncertainty of 0.189. At Youyi Farm, the size of the fields was much larger than that in the other study areas; the spatial heterogeneity is thus relatively small and has a mean standard deviation of 0.413. The scaling difference is the smallest among all the study areas and has a mean value of 0.013.

Table 5 lists the statistical metrics of the fine-resolution LAI validation maps for Longkang Farm, Anhui Province. These statistics are for eight reference maps corresponding to two growth stages in 2017. The LAI for these eight reference maps is relatively large, ranging from 2.190 to 4.651, with a mean uncertainty of 0.685. The spatial heterogeneity is similar to that in the Henan study area, with a mean standard deviation of 1.528. The scaling difference has a mean relative value of 0.553.

The field measurements, published for public use, are available at Zenodo, 10.5281/zenodo.5091251. The dataset contains readme files, compressed files of the fine-resolution LAI maps, and files of statistics for the reference maps. The intermediate NDVI files and reference LAI maps derived using the U2 upscaling methods are also provided^65^.

## Technical Validation

### Performance of the semiempirical models

**Fig. 4:**
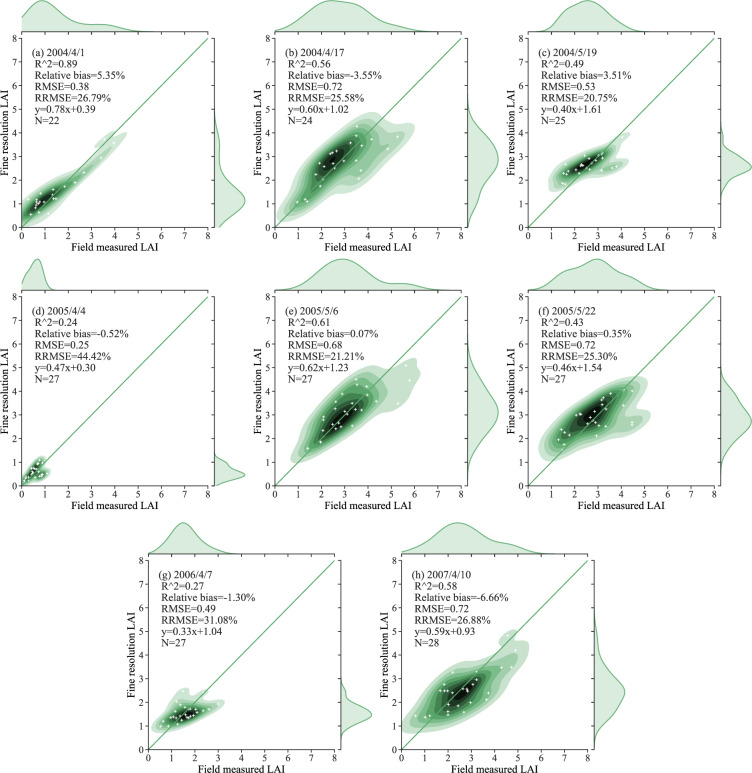
Comparison of the fine-resolution reference LAI and the field-measured data for wheat in different stages of growth in the Beijing study area.

**Fig. 5:**
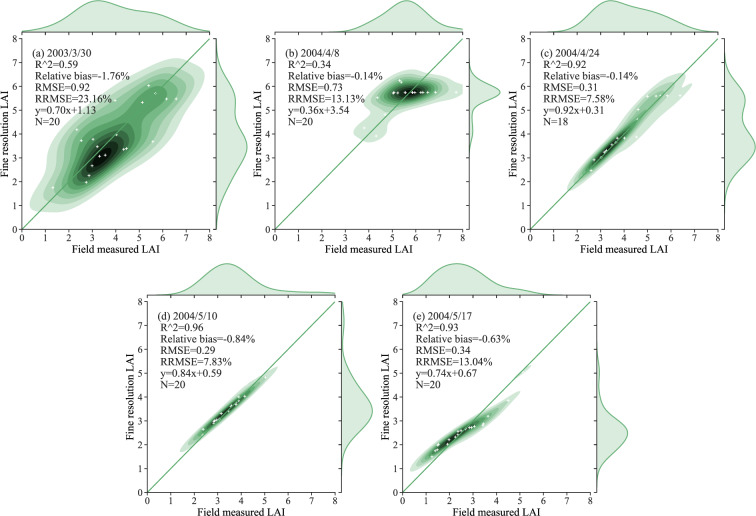
Comparison of the fine-resolution reference LAI values and the field-measured data for wheat in different stages of growth in Jiaozuo and Zhoukou, Henan Province.

**Fig. 6:**
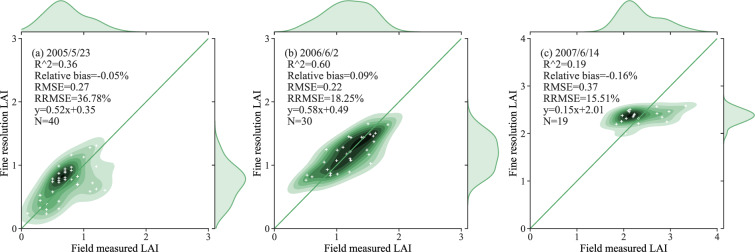
Comparison of the fine-resolution reference LAI values and the field-measured data for wheat and barley in different stages of growth at Youyi Farm, Heilongjiang Province.

**Fig. 7:**
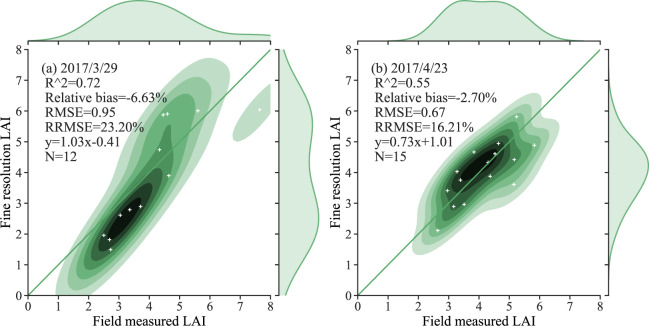
Comparison of the fine-resolution reference LAI values and the field-measured data for wheat in different stages of growth at Longkang Farm, Anhui Province.

The semiempirical NDVI-based models used to generate the fine-resolution reference LAI maps were validated using field measurements and the LOOCV method for the four study areas. This process is illustrated in Figs. 4–7. The results of a statistical comparison of the field-measured and generated LAI are also displayed in the figures.

In Figs. 4–7, the field-measured LAI values are compared with the LAI values derived by applying the semiempirical LAI model to Landsat TM/OLI SR data for the four study areas (Beijing, Henan, Heilongjiang, and Anhui). The results shown in Fig. 4 are characterized by slopes that are close to the 1:1 line, with RMSE values ranging from 0.25 to 0.72. As the results are displayed separately for each growth stage, the LAI values measured during the early growth stage have a wide distribution, with the result that the coefficient of determination for the regreening stage is low. Figure 5 displays the relationship between the field-measured LAI and the predicted LAI values for the Henan test area based on the formal semiempirical model: in this case, the RMSE ranges from 0.31 to 0.92, and the RRMSE is less than 23.16%. Figure 6 shows a comparison of the field-measured and predicted LAI values for Youyi Farm, Heilongjiang Province. On May 5th, 2005, and June 6th, 2006, field measurements of both wheat and barley were performed at this site; the samples collected on June 14th, 2007, were of barley only. Since barley and wheat are crops with similar vegetation structures, the two crop types are not separated in this comparison. The RMSE for these data has a range of 0.22 to 0.37, and the RRMSE has a range of 18.25% to 36.78%. The plots displayed in Fig. 7 show the relationship between the field-measured and predicted LAI values for Longkang Farm, Anhui Province. The slopes here are close to the 1:1 line, and the RMSE has a range of 0.67 to 0.95.

### Validation of MODIS LAI

**Fig. 8:**
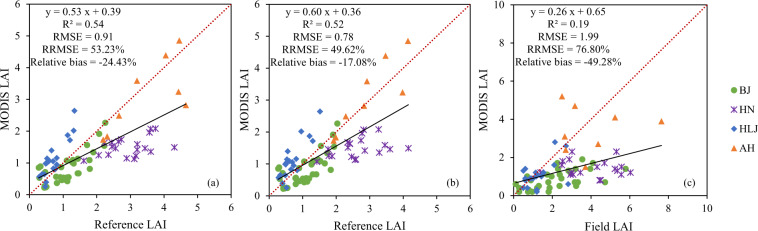
Validation results for the MCD15A2H LAI products obtained by applying (**a**) the U1 upscaling method to the LAI data from the 80 fine-resolution LAI maps and (**b**) the U2 upscaling method to LAI data from the 80 reference maps. (**c**) Validation results obtained using the corresponding field measurements.

The 80 reference LAI maps with a size of 3 km × 3 km derived from the two upscaling methods (Figure S8 in Supplementary Information) and the corresponding field LAI measurements were employed to validate the MODIS LAI V6 product (MCD15A2H) for the four study areas. The validation results are illustrated in Fig. 8 and Table 6.

**Table 6:** Validation metrics for the MODIS LAI product using data from the fine-resolution reference LAI maps and field-measured LAI values in the four study areas. R^2^: coefficient of determination, RMSE: root mean square error, RRMSE: relative RMSE, RB (relative bias): ratio of the difference in the MODIS and fine-resolution LAI to the fine-resolution LAI.

Study area	3 km × 3 km level comparison using validation dataset derived from U1 upscaling method				3 km × 3 km level comparison using validation dataset derived from U2 upscaling method				Direct field-to-pixel comparison			
	R^2^	RMSE	RRMSE	RB	R^2^	RMSE	RRMSE	RB	R^2^	RMSE	RRMSE	RB
All	0.54	0.91	53.2%	−24.4%	0.52	0.78	49.6%	−17.0%	0.19	1.99	76.8%	−49.3%
BJ	0.59	0.48	42.0%	−27.0%	0.54	0.45	41.5%	−24.0%	0.35	1.84	81.7%	−62.1%
HN	0.18	1.56	52.8%	−48.9%	0.13	1.29	48.9%	−43.0%	0.008	2.92	73.3%	−64.4%
HLJ	0.77	0.52	73.8%	56.9%	0.73	0.53	76.9%	59.8%	0.26	0.68	54.7%	−13.8%
AH	0.52	0.84	24.1%	−10.8%	0.77	0.55	18.8%	6.0%	0.004	2.03	50.8%	−13.6%

In Fig. 8(a), the fine-resolution reference LAI maps (30 m) derived from Eq. (4) were compared with the MODIS LAI in the range of 3 km × 3 km, which refers to the U1 upscaling method. To investigate how the scaling difference contributes to the discrepancies between the fine-resolution maps and the coarse-resolution products, the reference LAI maps at 500 m resolution were obtained based on the ‘average first and then invert’ (U2 upscaling) method with a size of 3 km × 3 km (as described in Figure S8). These reference LAI maps at 500 m resolution were compared with the MODIS LAI, as illustrated in Fig. 8(b). In addition, the field LAI measurements were directly compared with the corresponding MODSI LAI, as illustrated in Fig. 8(c).

The results illustrated in Fig. 8(a) indicate that the MODIS LAI values are underestimated in comparison to the fine-resolution reference LAI data in the range of 3 km × 3 km, especially in the case of the Henan study area. Table 6 shows that the accuracy of the MODIS LAI product varies among the study areas: the values are severely underestimated for crops in Beijing, Henan, and Anhui (relative bias = –27.0%, –48.9%, and –10.8%, respectively), whereas the values are overestimated for the crops with a black soil background in Heilongjiang Province (relative bias = 56.9%).

Due to the existence of surface heterogeneity, applying the model developed with 30 m data to 500 m data could result in some discrepancies. Since coarse-resolution LAI should be equal to aggregated fine-resolution LAI in the absence of scaling errors, validation using the reference LAI derived from the U2 method will result in artificially high accuracy^60^. However, by comparing the validation results from the U1 and U2 methods, the error due to the scale effect inherent to the coarse-resolution product can be at least partly quantified. In Fig. 8(b), the results gave an RMSE of 0.78 against the value of 0.91 that was obtained by applying the U1 (‘invert first and then average’) upscaling method to the reference LAI dataset in Fig. 8(a), which indicates that the scaling difference also contributes to the error in the coarse-resolution MODIS LAI product. When the scaling difference was taken into consideration and compensated for by applying the U2 upscaling method to the reference LAI dataset, the underestimates for the Beijing, Henan, and Anhui areas were reduced, giving relative biases of −24.0%, −43.0%, and 6.0%, respectively, compared with –26.9%, −48.9%, and −10.8% in Fig. 8(a), respectively. In terms of the accuracy of MODIS LAI in Heilongjiang, since the land cover in Heilongjiang is relatively uniform, the mean scaling difference among the four study areas is lowest, and the RMSE and relative bias thus slightly increased from 0.52 to 0.53 and 56.9% to 59.8%, respectively. A direct comparison with the field measurements (Fig. 8(c)) produced much higher uncertainties (RMSE = 1.99, RRMSE = 76.8%, relative bias = −49.3%) than were found by using the upscaled reference LAI dataset.

In this study, a highly accurate fine-resolution LAI dataset for Chinese croplands that could be used as a reference for coarse-resolution LAI products was derived from field measurements and fine-spatial-resolution satellite imagery (Landsat-5 TM and Landsat-8 OLI data). A semiempirical statistical model based on the Beer–Lambert law was used to derive fine-resolution LAI data that could be used for validation of the coarse-resolution LAI product at each growth stage. The parameters of each semiempirical model were estimated using the field LAI at each growth stage based on the curve-fitting algorithm and LOOCV approach. During this procedure, the performance of each semiempirical model was also investigated. Finally, eighty fine-resolution reference LAI maps with a size of 3 km × 3 km were generated for the study areas in four Chinese provinces. This fine-resolution reference LAI dataset was applied to assess the accuracy of MODIS LAI among these four study areas using the U1 upscaling method. The MODIS LAI was also compared to the reference LAI generated using the U2 upscaling method, through which the error due to the scale effect inherent to the coarse-resolution LAI product can be partly quantified. The direct comparison of the LAI data collected in the field and MODSI LAI showed considerable uncertainty. Therefore, this study contributes to the validation of remote sensing LAI products by providing a set of fine-resolution reference LAI datasets based on destructive sampling methods and highlights the importance of using a fine-resolution reference LAI dataset based on direct field measurements. Such a dataset can bridge the gap between field measurements and coarse-resolution pixel data.

## Supplementary information

Supplementary Information

## Data Availability

In the data repository^65^, the readme files explain the location of the files and folders. All raw measurements records can be found in one Excel sheet. All the field data and satellite images were processed and analysed in IDL and Python. The source codes are available at the Github. https://github.com/BowenSong123/Code.

## Online-only Table

**Online-only Table 1:** Details of the LAI field measurements and selected satellite imageries in the study areas (BJ: Beijing, HN: Henan, HLJ: Heilongjiang, AH: Anhui). “\” means there is no suitable satellite imagery.

Study area		Date of field measurement	No. of samples	Date of selected satellite imagery	Crops
BJ		2004/4/1	22	2004/4/1	wheat
		2004/4/15	24	2004/4/17	
		2004/4/28	22	\	
		2004/5/19	25	2004/5/19	
		2004/6/3	25	\	
		2005/4/3	27	2005/4/4	
		2005/4/13	27	\	
		2005/4/22	27	\	
		2005/5/9	27	2005/5/6	
		2005/5/23	27	2005/5/22	
		2006/4/7	27	2006/4/7	
		2006/4/19	27	\	
		2006/4/27	27	\	
		2006/5/10	27	\	
		2007/4/9	28	2007/4/10	
		2007/5/12	28	\	
HN	Jiaozuo	2003/3/22	20	\	wheat
		2003/3/30	20	2003/3/30	
		2003/4/7	20	\	
	Zhoukou	2004/4/7	20	2004/4/8	
		2004/4/24	18	2004/4/24	
		2004/5/10	19	2004/5/10	
		2004/5/18	20	2004/5/17	
HLJ		2005/5/23	20	2005/5/23	wheat
		2005/6/13	20	\	
		2005/6/24	20	\	
		2006/5/25	10	2006/6/2	
		2006/6/30	10	\	
		2006/7/12	10	\	
		2005/5/23	20	2005/5/23	barley
		2005/6/13	20	\	
		2005/6/24	20	\	
		2006/5/25	20	2006/6/2	
		2006/6/30	20	\	
		2007/6/12	19	2007/6/14	
		2007/6/22	20	\	
		2005/7/2	20	\	paddy rice
		2005/8/5	20	\	
		2005/9/6	20	\	
		2006/6/30	20	\	
		2006/7/27	20	\	
		2006/8/25	20	\	
		2007/7/6	20	\	
		2007/7/2	20	\	soybean
		2007/7/17	20	\	
AH		2017/3/26	12	2017/3/29	wheat
		2017/4/21	15	2017/4/23	
		2017/8/20	20	\	paddy rice
