# Supplementary material for: ValLAI_Crop, a validation dataset for coarse-resolution satellite LAI products over Chinese cropland
Source: Sci Data. 2021 Sep 20;8:243. doi: 10.1038/s41597-021-01024-4 (PMC8452658; doi:10.1038/s41597-021-01024-4)
Supplement: Supplementary file 1 — Supplementary Information [file 41597_2021_1024_MOESM1_ESM.pdf]

Table of contents

Determination of scaling difference using two different upscaling methods 6

Figure S1 2

Figure S2 3

Figure S3 3

Figure S4 4

Figure S5 4

Figure S6 5

Figure S7 5

Figure S8 6

Figure S9 7

Figure S10 9

Figure S11 10

Figure S12 10

Figure S13 11

Table S1 2

Table S2 3

Table S3 5

References 11

Figure S1. Location and vegetation type of the study areas in (b) Beijing, (c) Youyi farm in Heilongjiang province, (d) Longkang farm in Anhui province, (e) Zhoukou, Henan province, (f) Jiaozuo, Henan province, respectively.

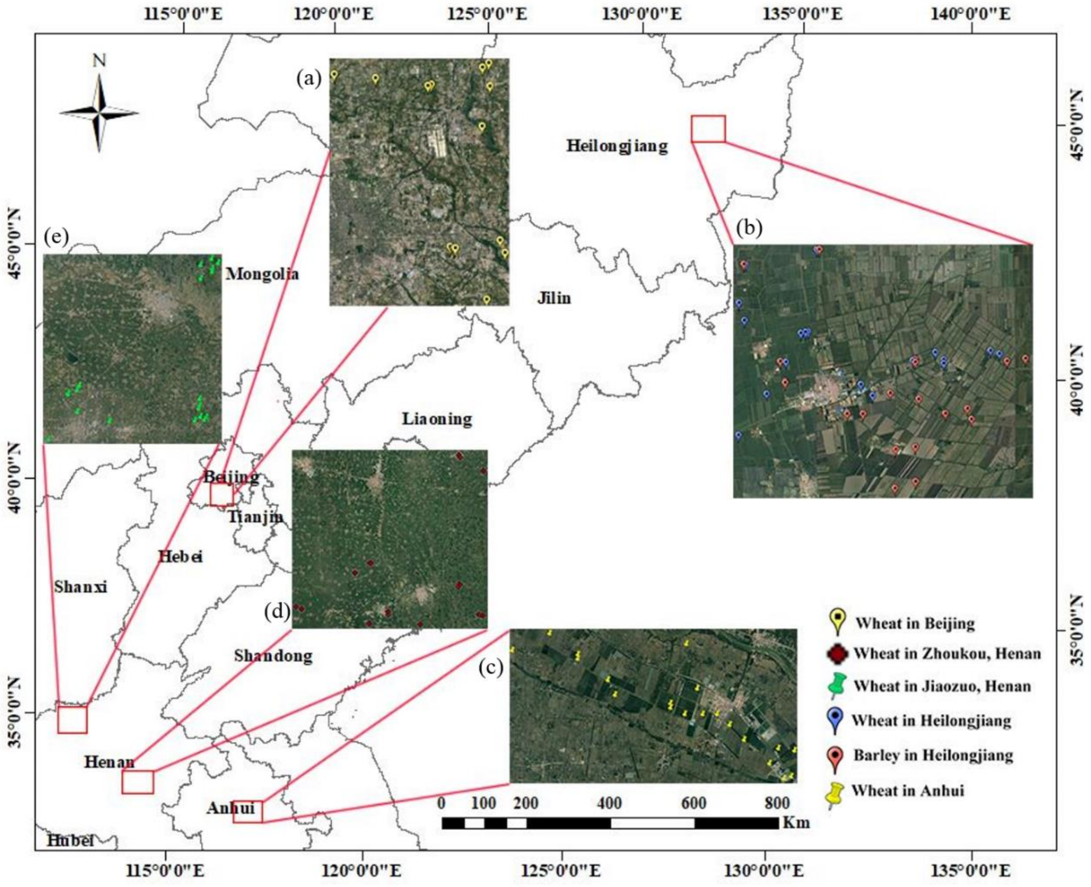

Table S1. The normalization function for Landsat TM red and near infrared bands in Beijing area. x refers to original band of TM, y refers to the corrected band of TM

Date	Red Band	NIR Band
2004/4/01	$y = 0.9040 \times x$	$y = 1.0535 \times x$
2004/4/17	$y = 0.946 \times x - 0.005$	$y = 0.897 \times x - 0.005$
2004/5/19	$y = 1.0684 \times x$	$y = 0.9935 \times x$
2005/4/04	$y = 0.948 \times x - 0.016$	$y = 0.991 \times x - 0.002$
2005/5/06	$y = 0.888 \times x + 0.021$	$y = 1.0455 \times x$
2005/5/22	$y = 0.934 \times x + 0.003$	$y = 1.1048 \times x$
2006/4/07	$y = 0.856 \times x + 0.014$	$y = 0.866 \times x + 0.035$
2007/4/10	$y = 0.8099 \times x$	$y = 1.0111 \times x$

Figure S2. The scatterplot of TM surface reflectance against MODIS surface reflectance at red-band reflectance in Beijing area

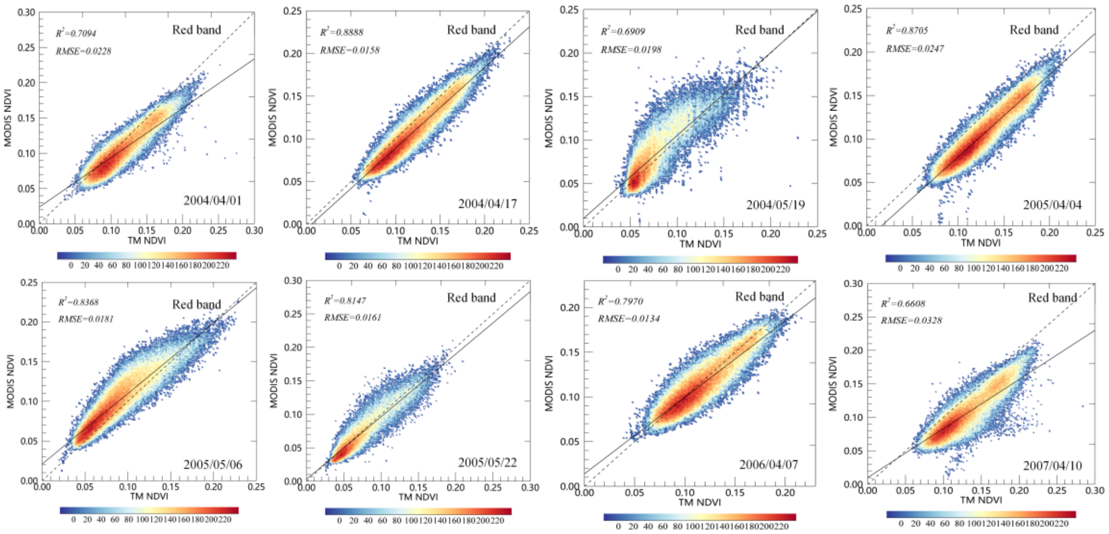

Figure S3. The scatterplot of TM surface reflectance against MODIS surface reflectance at near-infrared band reflectance in Beijing area

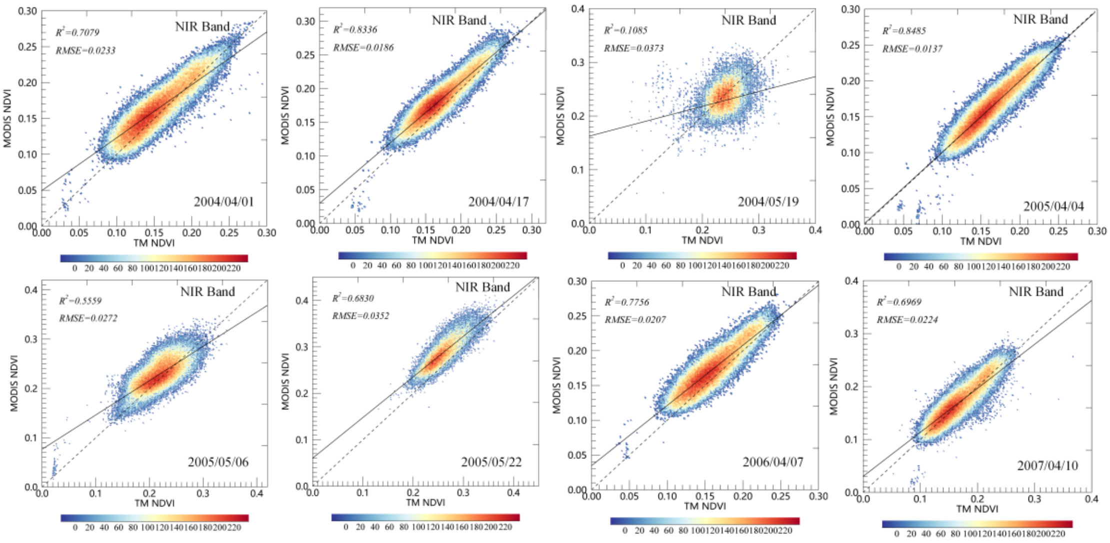

Table S2. The normalization function for Landsat TM red and near infrared bands in Henan area. x refers to original band of TM, y refers to the corrected band of TM

Date	Red Band	NIR Band
2003/3/30 (row:36)	$y = 0.9157 \times x$	$y = 0.9729 \times x$
2003/3/30 (row:37)	$y = 0.9844 \times x$	$y = 1.0239 \times x$
2004/4/08	$y = 1.3419 \times x$	$y = 1.2881 \times x$
2004/4/24	$y = 1.1945 \times x$	$y = 1.2641 \times x$
2004/5/10	$y = 1.0849 \times x$	$y = 1.1637 \times x$
2004/5/17	$y = 1.052 \times x + 0.015$	$y = 1.1909 \times x$

Figure S4. The scatterplot of TM surface reflectance against MODIS surface reflectance at red-band reflectance in Henan area

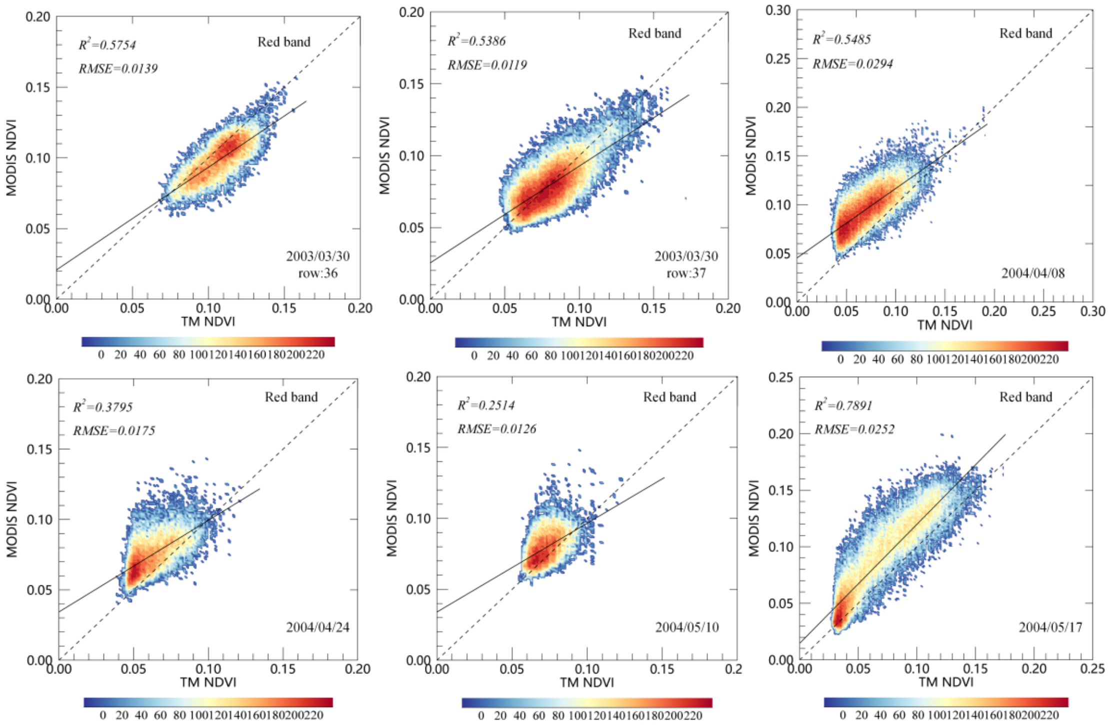

Figure S5. The scatterplot of TM surface reflectance against MODIS surface reflectance at near-infrared band reflectance in Henan area

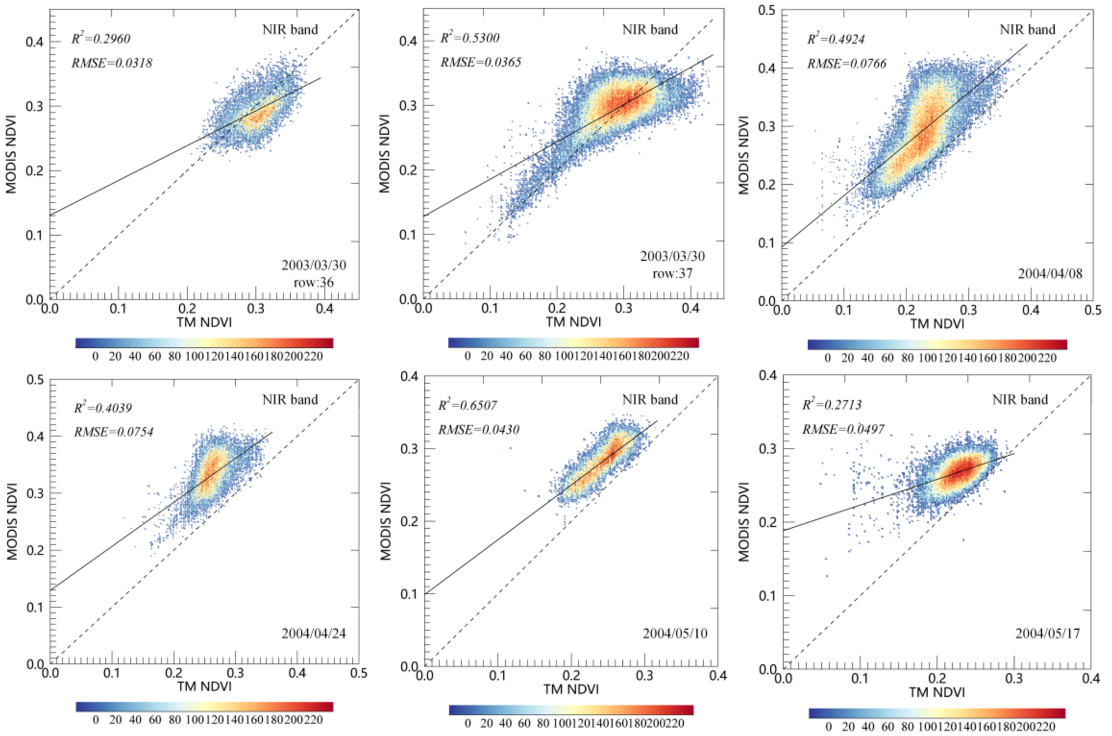

Table S3. The normalization function for Landsat TM red and near infrared bands in Heilongjiang area. x refers to original band of TM, y refers to the corrected band of TM

Date	Red Band	NIR Band
2005/5/23	$y = 0.9664 \times x$	$y = 1.2285 \times x$
2006/6/2	$y = 0.9305 \times x$	$y = 1.1092 \times x$
2007/6/14	$y = 1.0466 \times x$	$y = 1.015 \times x + 0.066$

Figure S6. The scatterplot of TM surface reflectance against MODIS surface reflectance at red-band reflectance in HLJ area

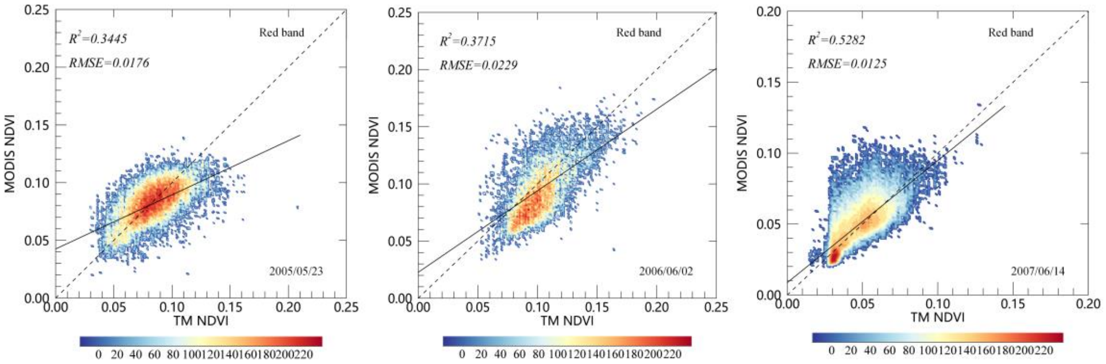

Figure S7. The scatterplot of TM surface reflectance against MODIS surface reflectance at near-infrared band reflectance in HLJ area

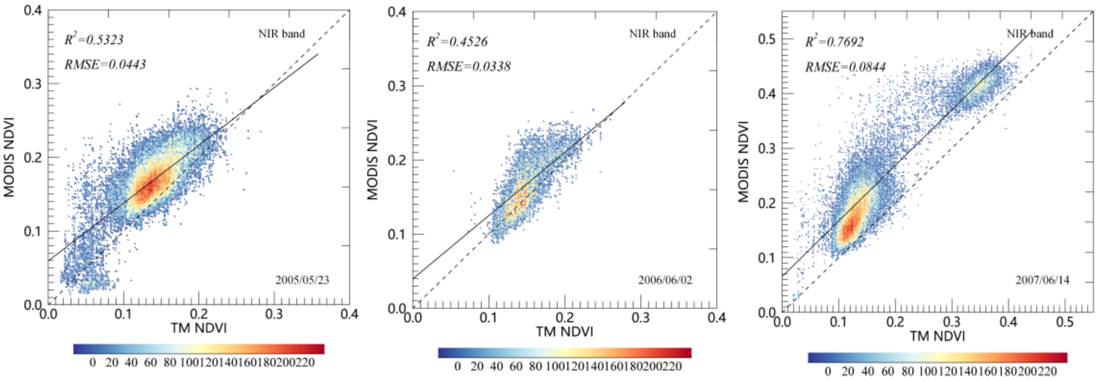

Determination of scaling difference using two different upscaling methods

The scale effect causes a degree of bias in remote sensing products with different spatial resolutions[26]. The scaling errors inherent to the coarse-resolution LAI product can be quantified by calculating the discrepancies caused by using two different upscaling methods, as illustrated in Figure S8.

Upscaling method (U1) is the so-called ‘invert first and then average’ method in which the fine-resolution NDVI is calculated first and the fine-resolution LAI is then retrieved based

on the semi-empirical NDVI-based model. The fine-resolution LAI maps are then aggregated (i.e. upscaled) to generate the coarse-resolution LAI. Upscaling method (U2) is the so-called 'average first and then invert' method. Using this method, the fine-resolution SR image is aggregated to a coarse-resolution image to derive the coarse-resolution NDVI. The semi-empirical NDVI-based model is then used to retrieve the coarse- resolution LAI. The difference of pixel value between the coarse-resolution LAI images obtained using the two different upscaling methods is defined as the spatial-scale difference.

According to an investigation by Liu et al. (2014) and Chen et al. (2002), the scale effect can produce underestimates in coarse-resolution LAI products in the case of pixels that are vegetation–soil mixtures and overestimates in the case of pixels that are vegetation–dark substrate (e.g., water or black soil) mixtures[26,64]. If the coarse-resolution LAI product is validated using the 'coarse' data upscaled from the fine-resolution maps based on the U1 upscaling method, the error in the final product can be attributed to the scaling difference as well as errors due to the inversion algorithm used and the data quantification error. However, if the coarse-resolution product is validated using the 'coarse' data upscaled from the fine-resolution maps based on the U2 upscaling method, the scaling difference will be partly quantified.

Figure S8. Diagram showing two different methods of up-scaling the LAI product

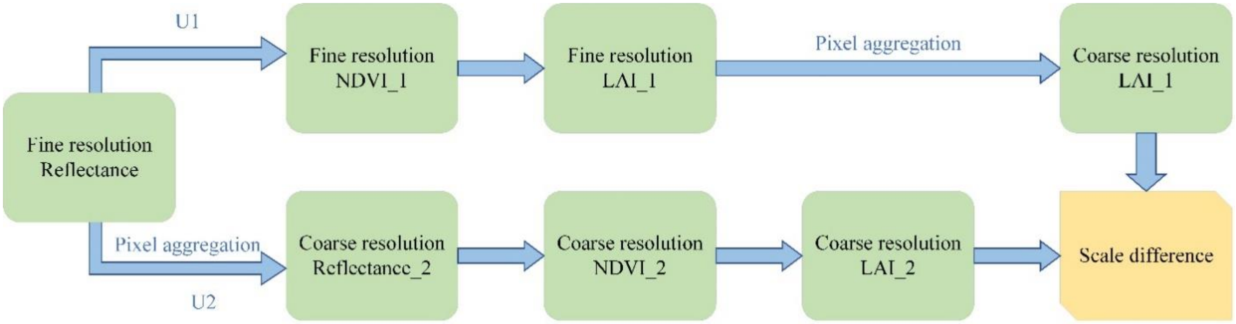

Figure S9. Fine resolution reference LAI maps derived for Beijing area. See Table 4 for more information about the fine resolution data. Noted that all the reference maps are 3 km × 3 km in size.

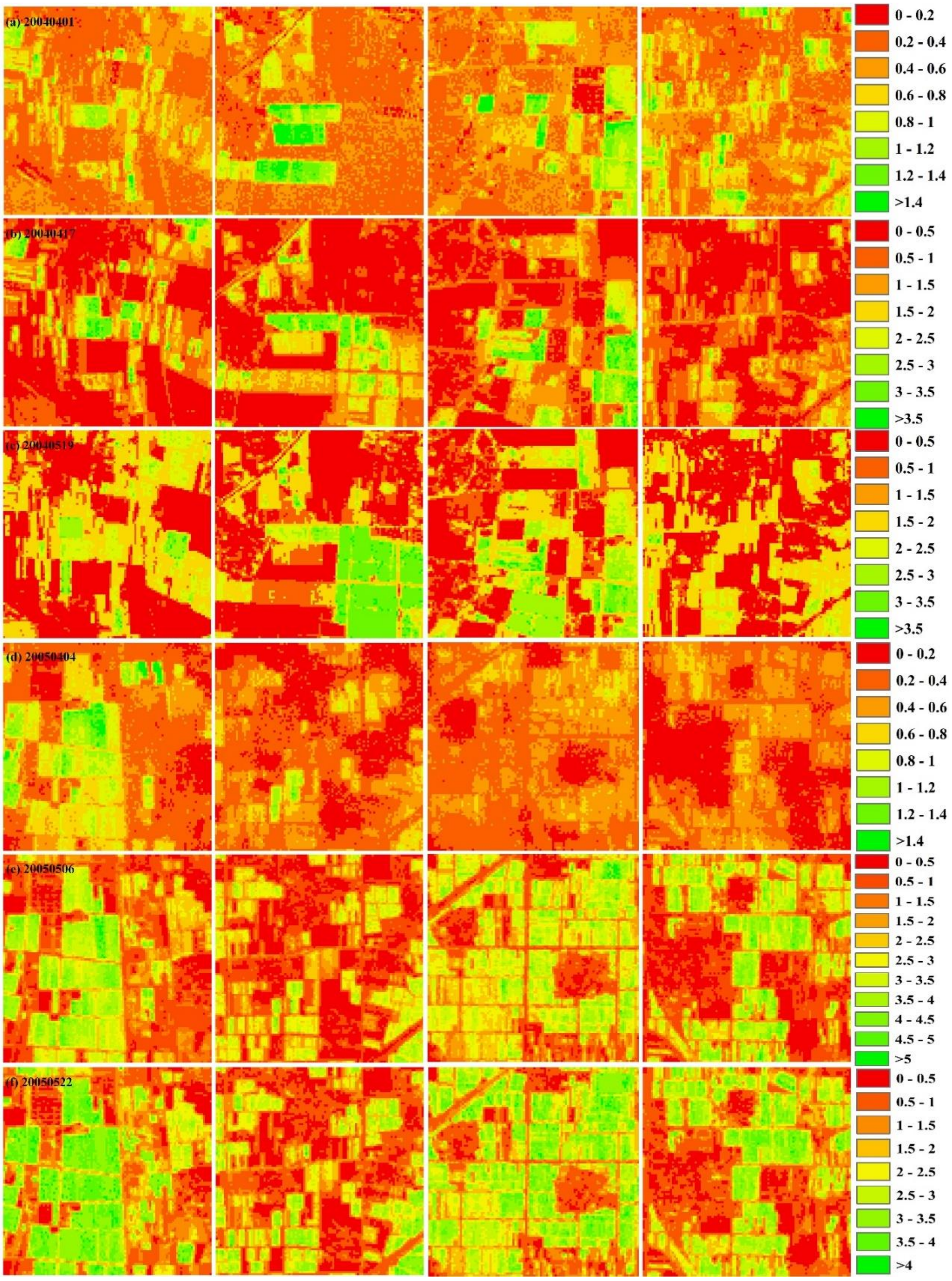

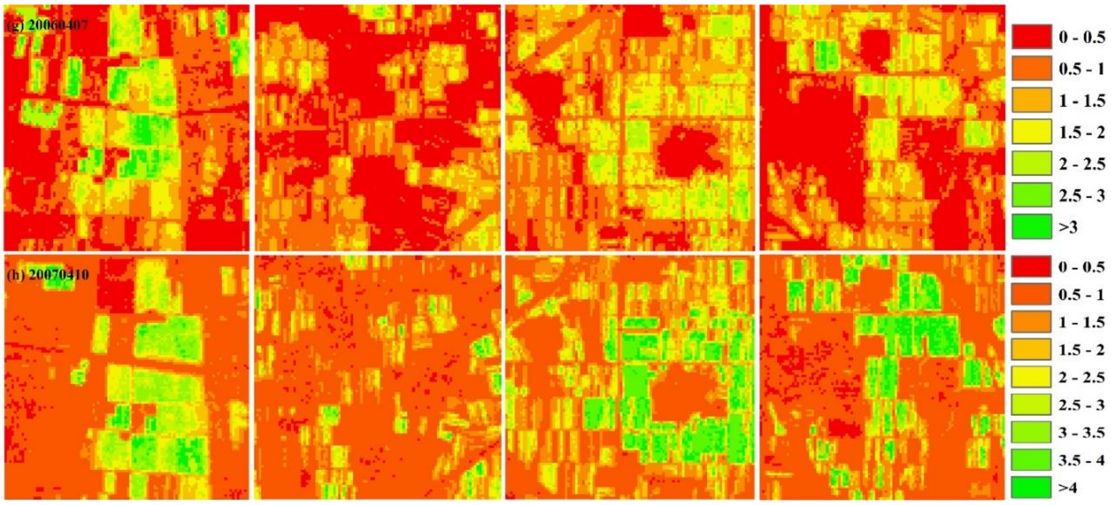
(g) 20060407
0 - 0.5
0.5 - 1
1 - 1.5
1.5 - 2
2 - 2.5
2.5 - 3
>3
(h) 20070410
0 - 0.5
0.5 - 1
1 - 1.5
1.5 - 2
2 - 2.5
2.5 - 3
3 - 3.5
3.5 - 4
>4

Figure S10. Fine resolution reference LAI maps derived for Henan area. See Table 5 for more information about the Fine resolution data. Noted that all the reference maps are 3 km × 3 km in size.

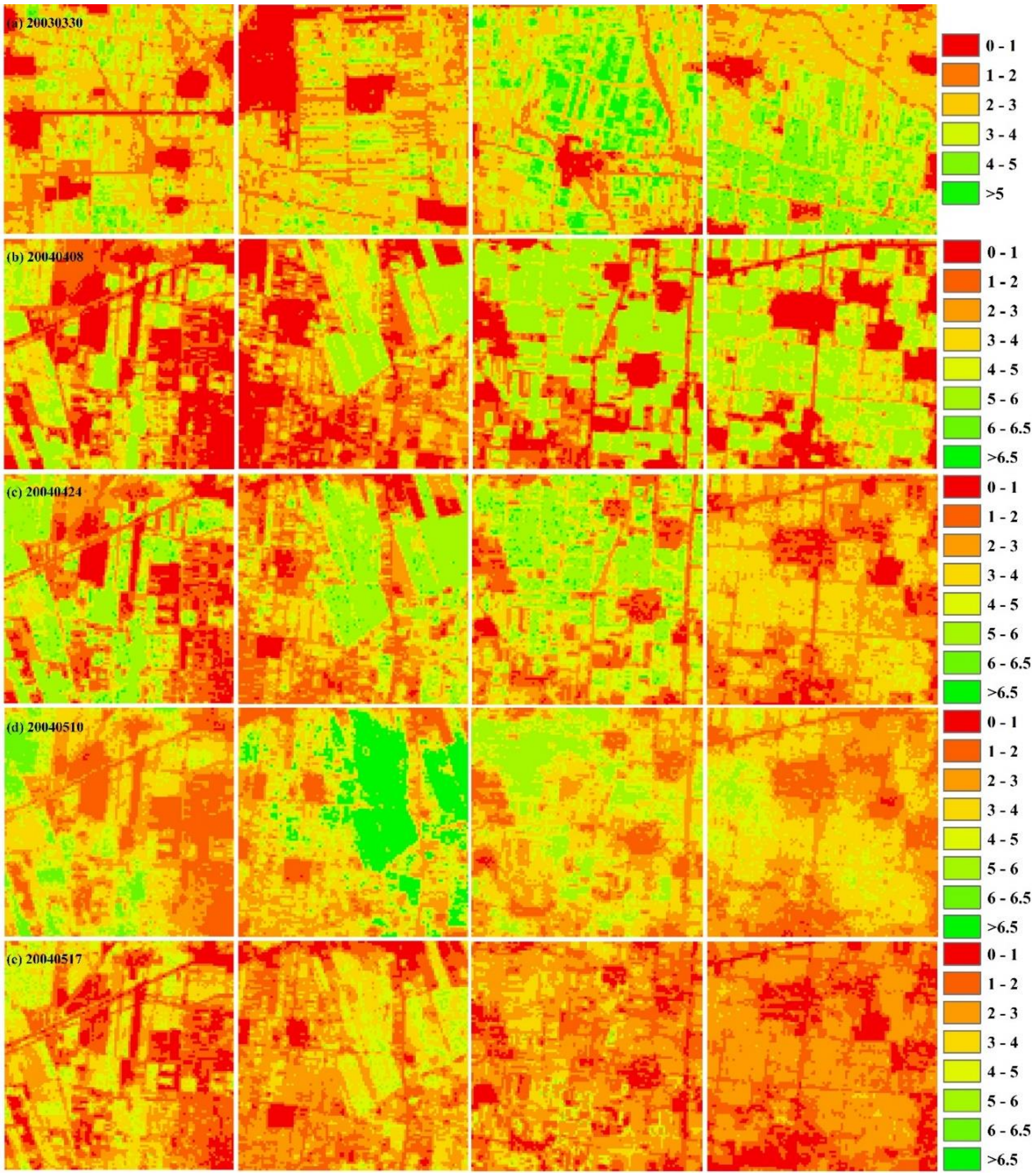

Figure S11. Fine resolution reference LAI maps derived for experimental site in Heilongjiang planted with wheat. See Table 6 for more information about the fine resolution data. Noted that all the reference maps are 3 km × 3 km in size.

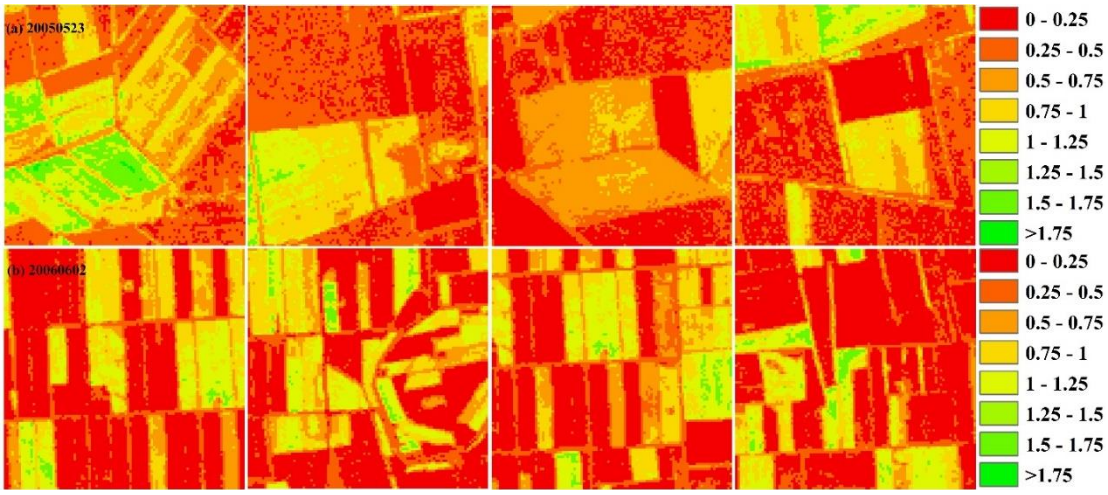

Figure S12. Fine resolution reference LAI maps derived for experimental site in Heilongjiang planted with barely. See Table 6 for more information about the fine resolution data. Noted that all the reference maps are 3 km × 3 km in size.

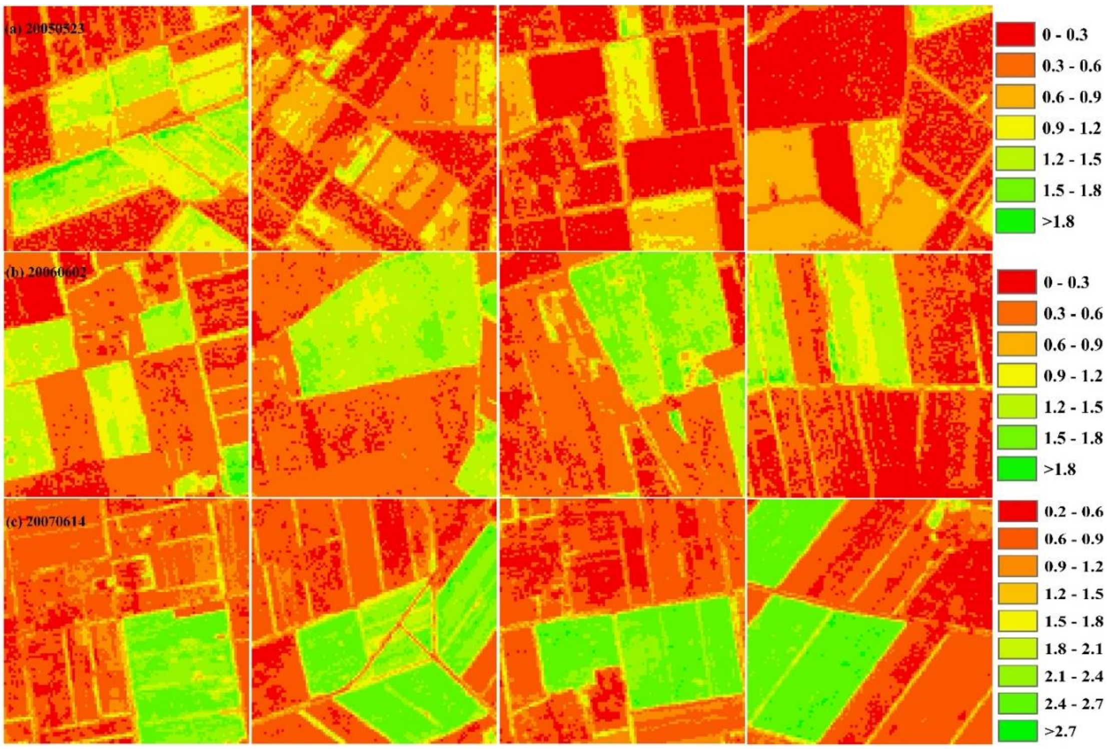

Figure S13 Fine resolution reference LAI maps derived for Anhui area. See Table 7 for more information about the fine resolution data. Noted that all the reference maps are 3 km × 3 km in size.

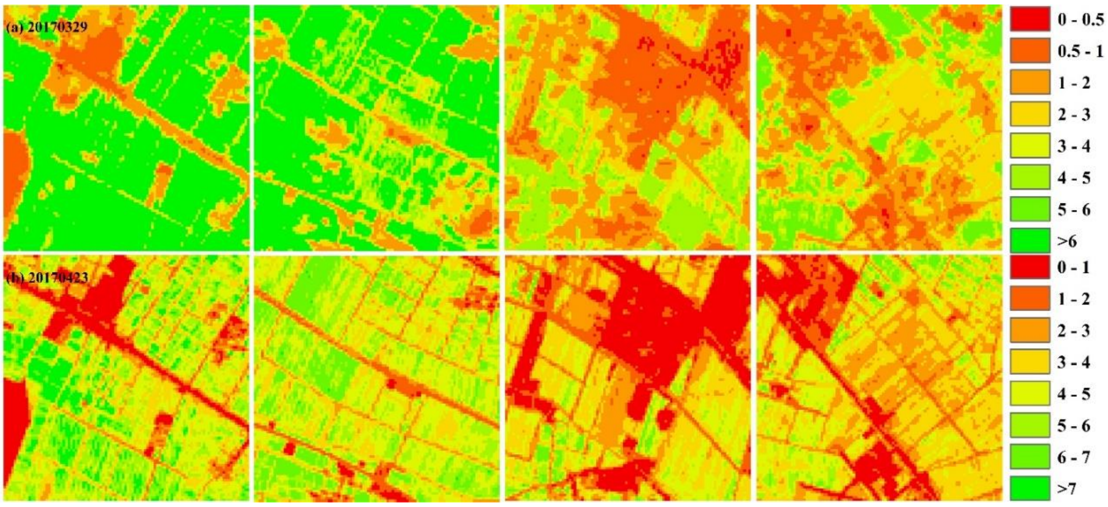

References:

26. Liu, L. Simulation and correction of spatial scaling effects for leaf area index (in Chinese). *J. Remote Sens* **18**, 1158-1168 (2014).

64. Chen, J. *et al.* Derivation and validation of Canada-wide coarse-resolution leaf area index maps using high-resolution satellite imagery and ground measurements. *Remote Sens. Environ* **80**, 165-184, (2002).
